# Emergence of a novel hybrid *mcr-1*-bearing plasmid in an NDM-7-producing ST167 *Escherichia coli* strain of clinical origin

**DOI:** 10.3389/fmicb.2022.950087

**Published:** 2022-08-24

**Authors:** Shuang Xia, Wei Wang, Jing Cheng, Tingting Zhang, Ziwei Xia, Xiaoyu Zhao, Yungang Han, Yonghong Li, Xiufang Shi, Shangshang Qin

**Affiliations:** ^1^Department of Medical Laboratory, Henan Provincial Chest Hospital, Zhengzhou, China; ^2^School of Pharmaceutical Sciences, Zhengzhou University, Zhengzhou, China; ^3^Key Laboratory of Advanced Drug Preparation Technologies, Ministry of Education, Zhengzhou University, Zhengzhou, China

**Keywords:** *Escherichia coli*, ST167, *mcr-1*, cointegrate plasmid, Is*26*

## Abstract

Colistin is considered as an antibiotic of ‘last resort’ for the treatment of lethal infections caused by carbapenem-resistant *Enterobacterales* (CRE), dissemination of plasmid-borne colistin resistance gene *mcr-1*, particularly into CRE, resulting in the emergence of strains that approach pan-resistance. A wide variety of plasmid types have been reported for carrying *mcr-1*. Among which, large IncHI2-type plasmids were multidrug-resistant (MDR) plasmids harbored multiple resistance determinants in addition to *mcr-1*. Herein, we characterized a novel hybrid IncHI2-like *mcr-1*-bearing plasmid in an NDM-7-producing ST167 *Escherichia coli* strain EC15-50 of clinical origin. Antimicrobial susceptibility testing showed *E. coli* EC15-50 exhibited an extensively drug-resistant (XDR) profile that only susceptible to amikacin and tigecycline. S1-PFGE, Southern hybridization and Whole-genome Sequencing (WGS) analysis identified a 46,161 bp *bla*_NDM-7_-harboring IncX3 plasmid pEC50-NDM7 and a 350,179 bp *mcr-1*-bearing IncHI2/HI2A/N/FII/FIA plasmid pEC15-MCR-50 in *E. coli* EC15-50. Sequence detail analysis revealed the type IV coupling protein (T4CP) gene was absent on pEC15-MCR-50, explaining that pEC15-MCR-50 was a non-conjugative plasmid. Comparative genetic analysis indicated the hybrid plasmid pEC15-MCR-50 was probably originated from pXGE1mcr-like IncHI2/HI2A/N plasmid and pSJ_94-like IncFII/FIA plasmid, and generated as a result of a replicative transposition process mediated by IS*26*. Currently, the prevalent *mcr-1-*carrying IncHI2 plasmids were rarely reported to be fused with other plasmids. The identification of the novel hybrid plasmid pEC15-MCR-50 in this study highlighted the importance of close surveillance for the emergence and dissemination of such fusion MDR plasmids, particularly in NDM-producing *Enterobacterales*.

## Introduction

Carbapenem-resistant *Enterobacterales* (CRE) strains, which exhibited multi-drug resistant or extensively drug resistant patterns and were associated with high mortality rates, were regarded as a serious threat for public health (Centers for Disease Control and Prevention, 2019). Given that less effective treatment options left for treating infections caused by CRE, colistin that belongs to cationic antimicrobial peptides with neurotoxic effects has been used as a last resort of antibiotic against CRE in clinic. Unfortunately, emergence and global dissemination of plasmid-borne colistin resistance gene *mcr-1* that encodes phosphoethanolamine transferase among *Enterobacterales*, especially in CRE, led to the emergence of potentially untreatable strains.

Since the first mobile colistin resistance (*mcr*) gene, *mcr-1*, was identified in *Escherichia coli* in 2016 from China, more *mcr* variants (*mcr-1* to *mcr-10*) were reported in different species of *Enterobacterales* exhibiting MDR phenotypes isolated from animals, food samples and clinical settings origin worldwide, and *mcr-1* was the most frequently detected and globally spread variant (Wang et al., 2018, 2020). Various *mcr-1*-encoding plasmids belonging to IncI2, IncX4, IncP, IncY, IncFII, IncN, IncQ and IncHI2 incompatibility groups have been described as the vector facilitates the dissemination of *mcr-1* (Ye et al., 2016; Wang et al., 2018). Among which, IncI2, IncHI2 and IncX4 were the most common plasmid types (Wu et al., 2018; Matamoros et al., 2020). Unlike these two types of small plasmids (IncI2: ~65 kb, IncX4: ~33 kb) that only carried a single resistance gene *mcr-1*, IncHI2 type plasmids with large size (> 200 kb) always harbored multiple resistance determinants beside *mcr-1* (Tang et al., 2020). In addition, hybrid *mcr-1*-bearing plasmids have also been described, such as pCQ02-121, a transferable IncX3/X4 hybrid plasmid co-harboring *mcr-1* and *bla*_NDM-5_ (Sun et al., 2016). Recently, the generation of hybrid and cointegrate *mcr-1* plasmids mediated by IS*26* or IS*CR2* were found in the process of conjugation (He et al., 2019; Li et al., 2020), promoting the dissemination of resistance genes and extending the resistance profile and the host spectrum for the fusion plasmid, which poses an enormous threat to public health.

Herein, we characterized a hybrid *mcr-1*-bearing plasmid containing IncFII, IncFIA, IncHI2, IncHI2A and IncN replicons in an NDM-7-producing extensively drug resistant (XDR) ST167 *E. coli* clinical isolate. Based on its structural features, we proposed a putative IS*26*-mediated formation model of this novel IncHI2/HI2A/N/FII/FIA hybrid plasmid.

## Materials and methods

### Bacterial isolation, antimicrobial susceptibility testing and PCR detection

*Escherichia coli* EC15-50 was obtained from the sputum culture of a 44-year-old male patient hospitalized in respiratory intensive care unit (RICU) at a tertiary hospital in Zhengzhou city in 2015. This isolate was identified by using the Vitek 2 system (bioMérieux, France) and 16S rDNA gene sequencing. Antimicrobial susceptibility testing was determined by using the broth microdilution method and agar dilution method (for fosfomycin) according to the Clinical and Laboratory Standards Institute guidelines (CLSI, 2020) and the European Committee on Antimicrobial Susceptibility Testing (EUCAST).^1^ *E. coli* ATCC25922 was used as the quality-control strain. PCR was used to detect the presence of the colistin resistance gene *mcr* and carbapenemase-encoding genes, including *bla*_NDM_, *bla*_IMP_, *bla*_OXA-48-like_, *bla*_VIM,_ and *bla*_KPC_, with primers described previously (Qin et al., 2014).

### S1-PFGE and Southern blotting

To detect the location of the *bla*_NDM-7_ and *mcr-1* gene, whole-cell genomic DNA of the *E. coli* EC15-50 isolate was digested by S1 nuclease (TaKaRa, Dalian, China) in agarose gel plug and then separated by pulsed-field gel electrophoresis (PFGE) under the conditions reported previously (Qin et al., 2014). The location of the *bla*_NDM-7_ and *mcr-1* was indicated by Southern hybridization using a digoxigenin-labeled *bla*_NDM-7_ and *mcr-1* gene fragment probe, respectively, using a DIG-High Prime DNA Labeling and Detection Starter Kit II (Roche Diagnostics, Basel, Switzerland) according to the manufacturer’s instructions.

### Whole-genome sequencing (WGS) and bioinformatics analysis

Whole-genome DNA of *E. coli* EC15-50 was extracted with the Wizard genomic DNA purification kit (Promega, Madison, WI, United States) and then sequenced using the Illumina MiSeq platform (San Diego, CA, United States) and the Pacific Biosciences RS II platform (Pacific Biosciences, Menlo Park, CA, United States). Sequencing reads were assembled into scaffolds using GS *De Novo* Assembler software (version 3.0). The prediction and annotation of ORFs were performed using Glimmer 3.02.^2^ Antimicrobial resistance genes (ARGs) were identified by using Resfinder (Bortolaia et al., 2020). Plasmid identified in this study was analyzed using PlasmidFinder^3^ and pMLST^4^ to investigate the replicons (Carattoli et al., 2014). Sequence alignments for the genetic environments of *mcr-1* were performed using BLAST.^5^

### Conjugation experiments

Conjugation assays were performed to assess the transferability of *mcr-1* gene according to the method described previously with minor modification (Wang et al., 2019). Briefly, approximately 1 × 10^8^ colony-forming units (CFU)/mL of the donor strain (EC15-50) and the rifampicin-resistant *E. coli* isolate EC600 as the recipient strain were mixed at a ratio of 1:4 on LB agar and cultured for 12 h. The mixtures were collected and then plated on an LB agar containing rifampicin (64 μg/ml) and colistin (1 μg/ml). All suspected transconjugants were verified by PCR amplification for *mcr-1*, and antimicrobial susceptibility testing.

### Plasmid stability testing

The stability of plasmid pEC15-MCR-50 was studied by passage in antibiotic-free Luria broth (LB) as previously described with minor modification (Sandegren et al., 2012). Briefly, the plasmid-containing *E. coli* EC15-50 were grown in 1 ml LB broth at 37°C for 12 h. The bacterial cells cultured for 12 h were washed by centrifugation and resuspended in 1 ml LB broth. Transfer 1 μl washed cells into 1 ml LB broth and incubate at 37°C for 12 h. For continuous culture, wash 1 μl cells into 1 ml LB broth once every 12 h, approximately 10 generations of growth per passage, for 10 consecutive days. The 200 generation strains were diluted and spread on LB agar plates. Then, 50 colonies were screened on LB agar plates supplemented with 2 μg/ml colistin, and the stability and integrity of the plasmid pEC15-MCR-50 carrying *mcr-1* gene across passages were investigated by PCR mapping technology. PCR primers were summarized in Supplementary Table S1.

## Results and discussion

### Characteristics of *Escherichia coli* EC15-50 co-harboring *bla*_NDM-7_ and *mcr-1*

**Figure 1:**
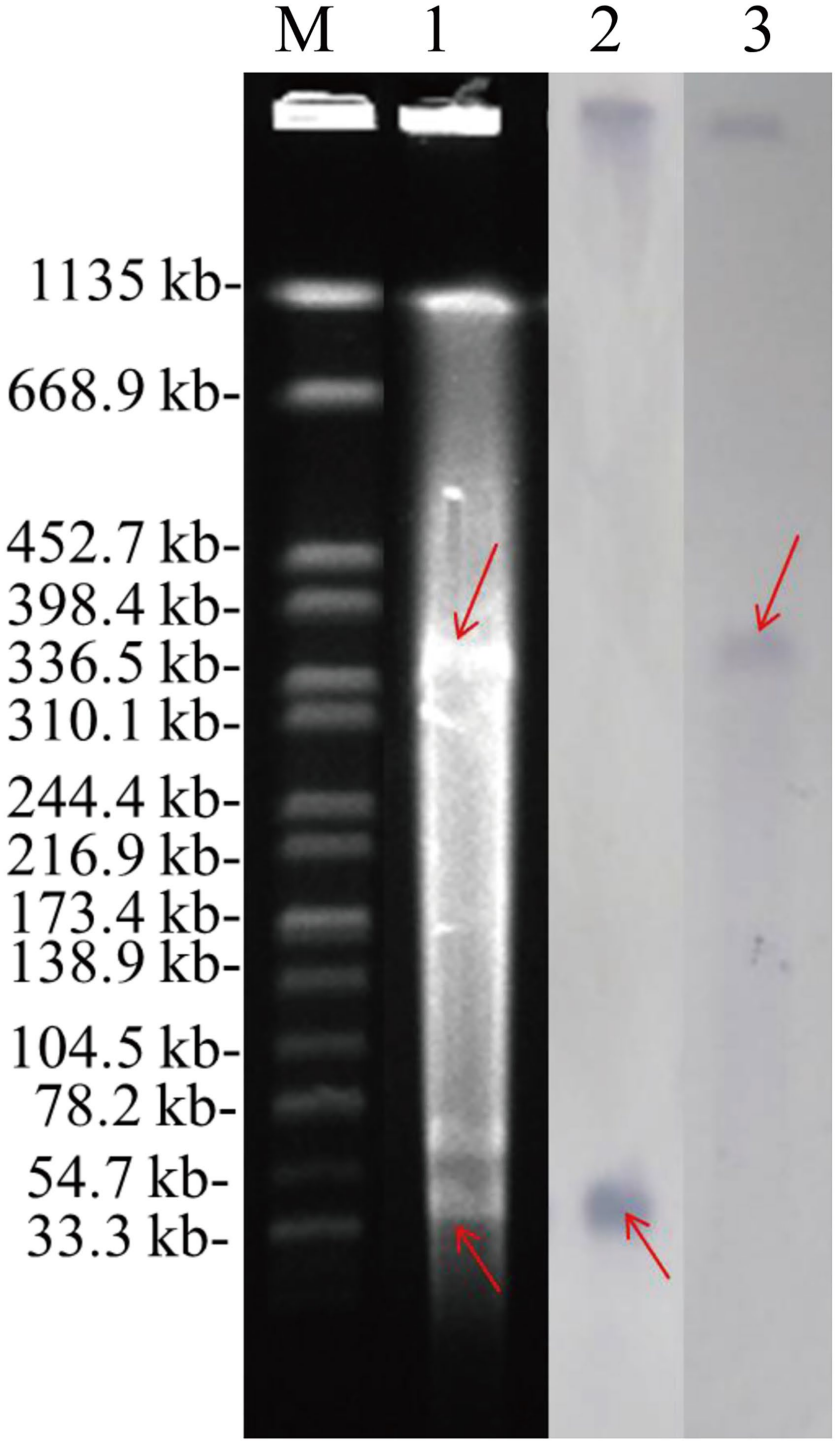
S1-PFGE and Southern hybridization of *mcr-1* and *bla*_NDM_ genes in the *Escherichia coli* strain EC15-50. The arrows indicate the locations of the plasmids hybridized to the *mcr-1* and *bla*_NDM_ probes. M, reference standard strain H9812 restricted with *XbaI*; Lane 1, *E. coli* EC15-50; Lane 2, the plasmid hybridized to the *bla*_NDM_-probe; Lane 3, the plasmid hybridized to the *mcr-1*-probe.

Clinical strain *E. coli* EC15-50 belonging to ST167 was recovered from the sputum culture of a 44-year-old male patient with acute lymphoblastic leukemia in 2015. *In vitro* antimicrobial susceptibility testing results showed that strain EC15-50 exhibited extensively drug resistant (XDR) profile for a wide range of antimicrobial agents, resistant to all cephalosporins, carbapenems, β-lactam/β-lactamase inhibitors, fluoroquinolones, gentamicin, chloroamphenicol, fosfomycin and doxycycline as well as colistin, but susceptible to amikacin and tigecycline (Table 1). PCR and sequencing based on carbapenems and colistin resistance profiles revealed the co-occurrence of *bla*_NDM-7_ *and mcr-1* gene in EC15-50. S1-PFGE and hybridization results further indicated the *bla*_NDM-7_ and *mcr-1* gene were located on separated plasmids with the sizes of ~45 kb and ~ 350 kb, respectively (Figure 1).

**Table 1:** Antimicrobial susceptibilities of strain *Escherichia coli* EC15-50.

Antimicrobial category	Antimicrobial agents	MIC^a^ (μg/mL)
		EC15-50
*Penicillins*	Ampicillin	>256^b^
Penicillins+β-lactamase inhibitors	Ampicillin/sulbactam	>256
*Antipseudomonal*		
Penicillins+β-lactamase inhibitors	Piperacillin/tazobactam	>256
*Non-extended spectrum*		
cephalosporins	Cefazolin	>256
*Extended-spectrum*		
Cephalosporins	Ceftazidime	>32
	Cefepime	>256
Carbapenems	Imipenem	>32
	Meropenem	>32
Monobactams	Aztreonam	>32
*Other antimicrobials*		
Fluoroquinolones	Ciprofloxacin	32
Aminoglycosides	Gentamicin	32
	Amikacin	≤2
Phenicols	Chloroamphenicol	>32
Phosphonic acids	Fosfomycin	1,024
Tetracyclines	Doxycycline	>32
Glycylcyclines	Tigecycline	≤0.5
Polymyxins	Colistin	4

a MIC, minimum inhibitory concentration.

b Resistance to antimicrobial agents showed in bold.

*E. coli* has been regarded as the predominant species in *Enterobacterales* for carrying *bla*_NDM_, as well as *mcr-1* gene, and co-occurrence of *bla*_NDM_ and *mcr* was most frequently detected in *E. coli* strains (Han et al., 2020). Among these *bla*_NDM_ and *mcr* positive strains, *bla*_NDM-5_ and *mcr-1* were the most common reported variants combination. However, the *bla*_NDM-7_ and *mcr-1* gene co-harbored *E. coli* have not been described. Our study represented the first report of clinical *E. coli* strain co-producing NDM-7 and MCR-1 that belongs to sequence type ST167. Notably, *E. coli* ST167 is classified as an internationally disseminated clonal lineage associated with the global spread of *bla*_CTX-M-15_ and *bla*_NDM_ genes. It has been considered as high-risk clone and is emerging associated with β-lactamases mainly in clinical samples origin throughout the world (Sánchez-Benito et al., 2017; Nukui et al., 2019; Xu et al., 2019). Dissemination of *mcr-1* within the successful epidemic clone may facilitate further spread of colistin resistance in β-lactamases-producing *E. coli* isolates.

### Identification of a novel hybrid *mcr-1*-bearing plasmid pEC15- MCR-50

**Figure 2:**
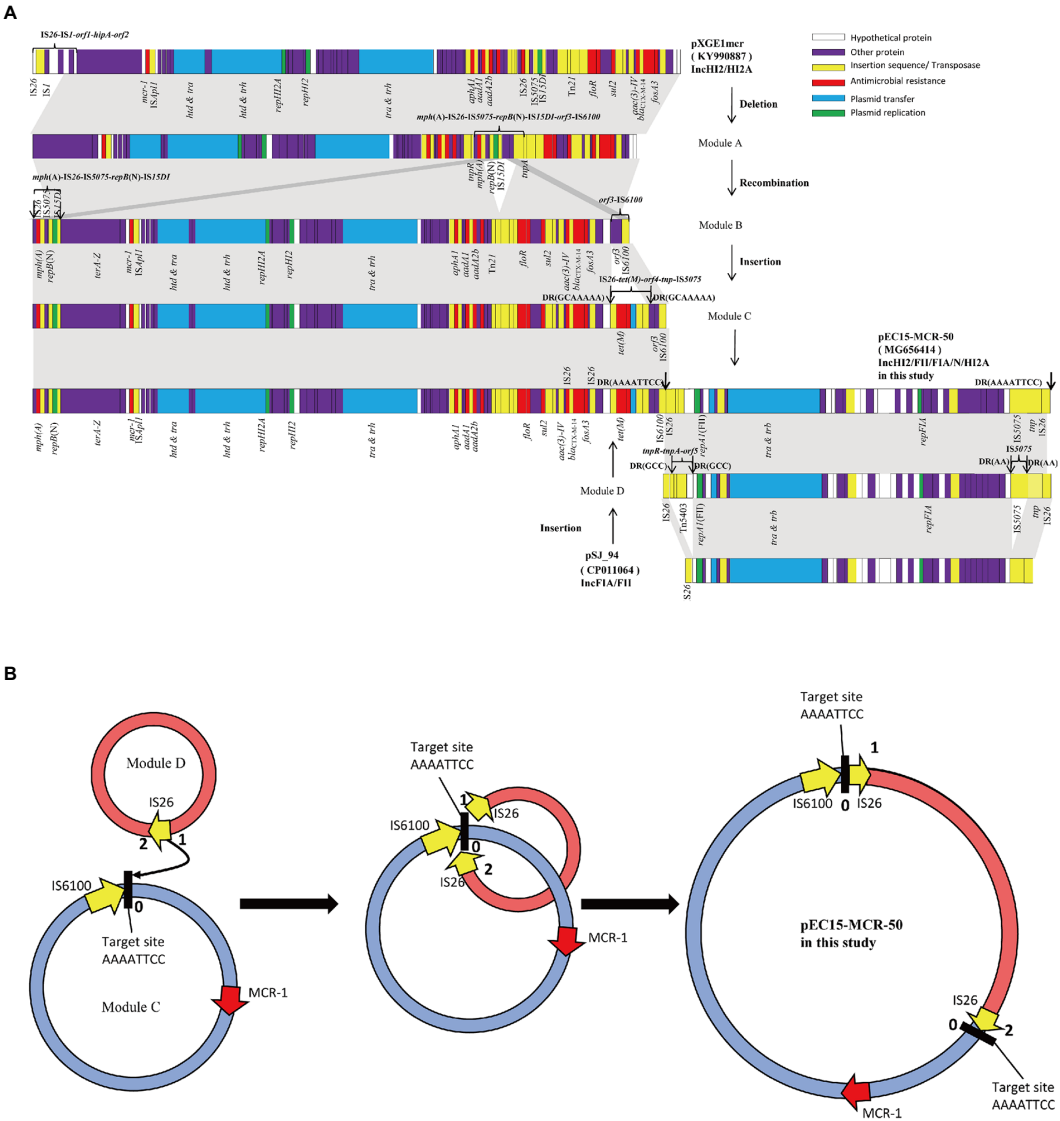
Schematic diagrams depicting the linear structures of the plasmid pEC15- MCR-50 and the proposed mechanism of plasmid fusion. **(A)** Schematic diagrams depicting the linear structure of the plasmid pEC15- MCR-50. White, purple, yellow, red, blue and green arrows indicate hypothetical protein, other genes, insertion sequence or transposase, resistance genes, plasmid transfer, and plasmid replication, respectively; The shaded area indicates 99–100% identity. **(B)** Proposed model for the formation of the fusion plasmid pEC15-MCR-50 mediated by IS*26*. Red arrow, *mcr-1* gene; Yellow arrow, IS*26*; Black bold string labelled with the number 0 represent the 8 bp target site (AAAATTCC).

Sequence analysis showed that the 46,161 bp *bla*_NDM-7_-harboring plasmid pEC50-NDM7 (KX470735), which was nearly identical to a reported plasmid pKpN01-NDM7 (CP012990) with a single nucleotide change from A to G at 31,701 bp, was a typical IncX3 plasmid carrying only one resistance gene *bla*_NDM-7_. However, the *mcr-1*-bearing plasmid pEC15-MCR-50 (MG656414), with a length of 350,179 bp and an average GC content of 48.0%, was a hybrid plasmid containing multiple replicons including IncFII, IncFIA, IncHI2, IncHI2A and IncN. ResFinder analysis identified a number of antimicrobial resistance genes in pEC15-MCR-50, including aminoglycosides resistance genes *aac(3)-IV*, *aph(3′)-Ia, aph(4)-Ia, aadA1 and aadA2b*, macrolides resistance gene *mph*(A), β-lactamases resistance gene *bla*_CTX-M-14_, phenicols resistance genes *cmlA1* and *floR,* tetracyclines resistance gene *tet*(M), fosfomycin resistance gene *fosA3*, sulfonamides resistance genes *sul2* and *sul3* in addition to colistin resistance gene *mcr-1* (Figure 2A). Interestingly, these resistance genes were distributed in three distinct regions of pEC15-MCR-50, a 3,673 bp *mcr-1*-containing cassette IS*Apl1-mcr-1-pap2* and a 6,867 bp *mph*(A)-harboring segment organized as IS*15DI-repB*(N)-IS*5075*-IS*26*-*mph*(A) were located in the backbone. However, the remaining resistance genes which were dispersed between multiple insertion sequences were located in the variable region. Additionally, genes responsible for plasmid maintenance including *parB, sopA* (plasmid partition) and *ccdAB* (toxin-antitoxin system) and conjugation and transfer (*tra*-relative genes) were also observed in the backbone.

Plasmid pEC15-MCR-50 failed to transfer from the donor strain *E. coli* EC15-50 to the recipient isolate *E. coli* EC600 in the conjugation assay. Sequence analysis by oriTfinder^6^ identified the *oriT* region, relaxase gene and T4SS gene cluster on plasmid pEC15-MCR-50, but the type IV coupling protein (T4CP) gene was absent. Given that the four components are necessary for conjugation in self-transmissible plasmids (Smillie et al., 2010), incomplete MOB module might be responsible for the non-conjugative profile of pEC15-MCR-50.

### Proposed formation mechanism of the plasmid pEC15-MCR-50

Based on the detailed sequence analysis of pEC15-MCR-50, we found it was a cointegrate plasmid with chimeric characteristics consisting of a ~243-kb pXGE1mcr (KY990877) -derived module (Module C) and a ~107-kb pSJ_94 (CP011064) -derived module (Module D; Figure 2A). Comparative genomic analysis between the hypothetical ancestor plasmid pXGE1mcr and the Module C sequence indicated that formation of this module was due to a series of genetic events. It is proposed that the deletion of a 12,747 bp of IS*26*-IS*1*-*orf1*-*hipA*-*orf2* in pXGE1mcr formed Module A. Subsequently, the genetic rearrangement of a 9,379 bp fragment organized as IS*6100*-*orf3*-IS*15DI*-*repB*(N)-IS*5075*-IS*26*-*mph*(A) immediately upstream of the *tnpA* gene and downstream of the *tnpR* gene, causing the insertion of macrolide resistance determinant containing part IS*15DI*-*repB*(N)-IS*5075*-IS*26*-*mph*(A) in the backbone region and the integration of the remnant part of the 2,512 bp fragment between the *tnpA* gene and the IS*15DI*, resulting in the formation of Module B. Finally, insertion of the IS*26*-*tet*(M)-*orf4-tnp-*IS*5075* element, conferring resistance to tetracyclines, as previously described in pHS13-1-IncHI2 (Liu et al., 2020a), upstream of the *orf3* gene in Module B formed Module C. On the other hand, Module D was proposed formation from an IncFIA/FII plasmid pSJ_94 (CP011064) by insertion of Tn*5403* (*tnpR*-*tnpA*-*orf5*) downstream of *repA1* flanked by a 3 bp direct repeat (GCC) and a 1,005 bp of IS*5075* upstream of the *tnp gene* at the end of the sequence flanked by a 2 bp direct repeat (AA), respectively.

The observed presence of an 8 bp direct repeat (AAAATTCC) bracketed the Module D segment indicated cointegrate formation is much more likely to be mediated by IS*26* element. Close inspection of the overall structure of pEC15-MCR-50, we proposed a possible model of cointegrate formation indicated by Figure 2B. In this model, IS*26* in Module D attacked the target site 0 (AAAATTCC) in *mcr-1*-harbored Module C and then replicative cointegrate formation occurred. Module D was incorporated into the downstream of IS*6100* in Module C creating the cointegrate pEC15-MCR-50 and resulting in an 8 bp target site duplications (TSD) and acquisition of an additional copy of IS*26*. Eventually, two direct repeats of the TSDs were present surrounding Module D.

IS*26* was regarded as a vital mobile element contributed to the dissemination and rearrangement of resistance determinants, as well as plasmid reorganization (Harmer and Hall, 2015; He et al., 2015; Wei et al., 2022). A previous study demonstrated that IS*26* was involved in recombination of an IncN1-F33: A-: B- plasmid and an *mcr-1*-carrying phage-like plasmid during conjugation process, facilitating the dissemination of *mcr-1* (He et al., 2019). Additionally, IS*26* was also reported to play an important role in generation of *bla*_NDM_ and *bla*_KPC_-bearing MDR cointegrate plasmids. A 59 kb cointegrate plasmid pT18 (CP017086) from *Proteus mirabilis* was likely to be generated by IS*26*-mediated cointegrate formation between the IncN-type plasmid pT211 (containing *bla*_KPC-2_) and the IncFII-33 plasmid (*bla*_TEM-1B_, *bla*_CTX-M-65_, *rmtB* and *fosA3*; Hua et al., 2020), and a 122 kb plasmid pL53T in the transconjugant of *E. coli* L53 was formed due to IS*26*-mediated fusion of pL53-4 (CP034737), a typical *bla*_NDM-5_-bearing IncX3 plasmid, and pL53-3 (CP034736), an IncFII-type plasmid harboring *fosA*, *bla*_CTX-M-55_ and *bla*_TEM-1_, during the conjugation process (Liu et al., 2020b). IS*26*-mediated plasmid recombination can not only lead to the emergence of large-size MDR plasmids but also accelerate the evolution of virulence plasmids. The entire *Salmonella* Enteritidis-specific virulence plasmid pSEN (HG970000) was observed to fuse with another IncHI2 MDR plasmid (KX518743) mediated by IS*26* to generate a cointegrate virulence–resistance plasmid pSE380T (KY401053; Wong et al., 2017). Similar to pSE380T, the 244 kb plasmid pCR-HvKP3TC-2_Vir-p3 (OM001475) was also found to be a fusion plasmid generated by IS*26*-mediated recombination of virulence plasmid pVir-CR-HvKP3 (MW598234) and plasmid p3-CR-HvKP3 (MW598238; Yang et al., 2022).

In addition to IS*26*, IS*CR2* has also been reported to reorganize the *mcr-1*-bearing large MDR plasmid and was responsible for *mcr-1*-bearing large fused MDR plasmids (Li et al., 2020). Compared with previous reported IS*26*-mediated N1-F33: A-: B- type *mcr-1*-bearing hybrid plasmid (MK419512), the HI2/HI2A/N/FII/FIA type plasmid pEC15-MCR-50 which possibly generated as a result of a replicative transposition process mediated by IS*26*, was identified as an MDR plasmid, carrying more resistance determinants. Although this plasmid could not be transferred by conjugation, it remained stable in strain in antimicrobial agent-free environment and could be served as a reservoir of multiple resistance genes. Based on the important role of IS*26* in mediating plasmid reorganization, further attention should be paid to focusing on this element in hybrid plasmids to trace the evolution of MDR or virulence plasmids.

### The stability of the plasmid pEC15-MCR-50

The serial passages and PCR mapping results showed that the plasmid pEC15-MCR-50 from *E. coli* EC15-50 could be retained at 100% over 200 generations of passage, revealing that the large fusion plasmid pEC15-MCR-50 remained stable in antimicrobial agent-free environment, and the resistance genes in this novel hybrid plasmid can be stably inherited.

## Conclusion

Polymyxin is regarded as the last line of defense in the clinical treatment of CRE infections. Emergence of *mcr-1* in NDM-producing *Enterobacterales* can seriously limit clinical treatment options. Our study identified and characterized an *mcr-1*-bearing IncHI2/HI2A/N/FII/FIA hybrid plasmid in NDM-7-producing XDR ST167-type *E. coli* of clinical origin. Detail analysis showed that IS*26* might be responsible for this *mcr-1*-bearing large fused plasmid. The formation of fusion plasmid was likely to maintain the stability of *mcr-1* gene, which may facilitate bacteria to adapt to the antibiotic stress. Close surveillance is urgently needed to monitor the emergence and dissemination of *E. coli* co-producing NDM and MCR-1, as well as such fusion MDR plasmids.

## Data availability statement

The datasets presented in this study can be found in online repositories. The names of the repository/repositories and accession number(s) can be found at: NCBI – KX470735, MG656414.

## Author contributions

SQ, XS, and YL designed the study. SX, WW, JC, and YH performed the experiments. TZ, JC, ZX, and XZ analyzed the bioinformatics data. SQ and JC wrote the manuscript. All authors contributed to the article and approved the submitted version.

## Funding

This work was supported by grant U2004125 from the National Natural Science Foundation of China (SQ), the Key R&D and Promotion Project of Henan Province (No. 212102310246, 2018010038) of China.

## Conflict of interest

The authors declare that the research was conducted in the absence of any commercial or financial relationships that could be construed as a potential conflict of interest.

## Publisher’s note

All claims expressed in this article are solely those of the authors and do not necessarily represent those of their affiliated organizations, or those of the publisher, the editors and the reviewers. Any product that may be evaluated in this article, or claim that may be made by its manufacturer, is not guaranteed or endorsed by the publisher.

## Footnotes

^1^ http://www.eucast.org

^2^ http://cbcb.umd.edu/software/glimmer/

^3^ https://cge.cbs.dtu.dk/services/PlasmidFinder/

^4^ https://cge.cbs.dtu.dk/services/pMLST/

^5^ https://blast.ncbi.nlm.nih.gov/Blast.cgi

^6^ https://tool-mml.sjtu.edu.cn/oriTfinder/oriTfinder.html

## References

[ref1] Bortolaia V. Kaas R. S. Ruppe E. Roberts M. C. Schwarz S. Cattoir V.. (2020). ResFinder 4.0 for predictions of phenotypes from genotypes. J. Antimicrob. Chemother. 75, 3491–3500. doi: 10.1093/jac/dkaa345, PMID: 32780112 PMC7662176

[ref2] Carattoli A. Zankari E. García-Fernández A. Voldby Larsen M. Lund O. Villa L.. (2014). In silico detection and typing of plasmids using PlasmidFinder and plasmid multilocus sequence typing. Antimicrob. Agents Chemother. 58, 3895–3903. doi: 10.1128/aac.02412-14, PMID: 24777092 PMC4068535

[ref3] Centers for Disease Control and Prevention. (2019). Antibiotic resistance threats in the United States. Available at https://stacks.cdc.gov/view/cdc/82532

[ref01] CLSI. (2020). Performance Standards for Antimicrobial Susceptibility Testing; 30th Informational Supplement. CLSI Document M100. Wayne, PA: CLSI.

[ref4] Han H. Liu W. Cui X. Cheng X. Jiang X. (2020). Co-existence of *mcr-1* and *bla*_NDM-5_ in an *Escherichia coli* strain isolated from the pharmaceutical industry, WWTP. Infect. Drug Resist. 13, 851–854. doi: 10.2147/idr.S245047, PMID: 32214831 PMC7083644

[ref5] Harmer C. J. Hall R. M. (2015). IS*26*-mediated precise excision of the IS26-aphA1a translocatable unit. mBio 6, e01866–e01815. doi: 10.1128/mBio.01866-15 26646012 PMC4676283

[ref6] He S. Hickman A. B. Varani A. M. Siguier P. Chandler M. Dekker J. P.. (2015). Insertion sequence IS26 reorganizes plasmids in clinically isolated multidrug-resistant bacteria by replicative transposition. MBio 6:e00762. doi: 10.1128/mBio.00762-15, PMID: 26060276 PMC4471558

[ref7] He D. Zhu Y. Li R. Pan Y. Liu J. Yuan L.. (2019). Emergence of a hybrid plasmid derived from IncN1-F33: A-: B- and *mcr-1*-bearing plasmids mediated by IS*26*. J. Antimicrob. Chemother. 74, 3184–3189. doi: 10.1093/jac/dkz327, PMID: 31360994

[ref8] Hua X. Zhang L. Moran R. A. Xu Q. Sun L. van Schaik W.. (2020). Cointegration as a mechanism for the evolution of a KPC-producing multidrug resistance plasmid in *Proteus mirabilis*. Emerg. Microbes Infect. 9, 1206–1218. doi: 10.1080/22221751.2020.1773322, PMID: 32438864 PMC7448864

[ref9] Li R. Lu X. Peng K. Liu Y. Xiao X. Wang Z. (2020). Reorganization of *mcr-1*-bearing large MDR plasmids resolved by nanopore sequencing. J. Antimicrob. Chemother. 75, 1645–1647. doi: 10.1093/jac/dkaa046, PMID: 32068848

[ref10] Liu Y.-y. Sun Y.-w. Sun H.-r. Luo X.-w. He D.-d. Wu H.. (2020a). Characterization of the IncHI2 plasmid pTW4 harboring *tet*(M) from an isolate of *Escherichia coli* ST162. J. Antibiot. 73, 876–880. doi: 10.1038/s41429-020-0337-y, PMID: 32528162

[ref11] Liu Z. Xiao X. Liu Y. Li R. Wang Z. (2020b). Recombination of NDM-5-producing plasmids mediated by IS*26* among *Escherichia coli*. Int. J. Antimicrob. Agents 55:105815. doi: 10.1016/j.ijantimicag.2019.09.019, PMID: 31600553

[ref12] Matamoros S. van Hattem J. M. Arcilla M. S. Willemse N. Melles D. C. Penders J.. (2020). Global phylogenetic analysis of *Escherichia coli* and plasmids carrying the *mcr-1* gene indicates bacterial diversity but plasmid restriction. Sci. Rep. 7, 15364. doi: 10.1038/s41598-020-58953-0, PMID: 29127343 PMC5681592

[ref13] Nukui Y. Ayibieke A. Taniguchi M. Aiso Y. Shibuya Y. Sonobe K.. (2019). Whole-genome analysis of EC129, an NDM-5-, CTX-M-14-, OXA-10- and MCR-1-co-producing *Escherichia coli* ST167 strain isolated from Japan. J. Glob. Antimicrob. Resist. 18, 148–150. doi: 10.1016/j.jgar.2019.07.001, PMID: 31295582

[ref14] Qin S. Fu Y. Zhang Q. Qi H. Wen J. G. Xu H.. (2014). High incidence and endemic spread of NDM-1-positive Enterobacteriaceae in Henan Province, China. Antimicrob. Agents Chemother. 58, 4275–4282. doi: 10.1128/aac.02813-13, PMID: 24777095 PMC4136005

[ref15] Sánchez-Benito R. Iglesias M. R. Quijada N. M. Campos M. J. Ugarte-Ruiz M. Hernández M.. (2017). *Escherichia coli* ST167 carrying plasmid mobilisable *mcr-1* and *bla*_CTX-M-15_ resistance determinants isolated from a human respiratory infection. Int. J. Antimicrob. Agents 50, 285–286. doi: 10.1016/j.ijantimicag.2017.05.005, PMID: 28599866

[ref16] Sandegren L. Linkevicius M. Lytsy B. Melhus Å. Andersson D. I. (2012). Transfer of an *Escherichia coli* ST131 multiresistance cassette has created a *Klebsiella pneumoniae*-specific plasmid associated with a major nosocomial outbreak. J. Antimicrob. Chemother. 67, 74–83. doi: 10.1093/jac/dkr405, PMID: 21990049

[ref17] Smillie C. Garcillán-Barcia M. P. Francia M. V. Rocha E. P. de la Cruz F. (2010). Mobility of plasmids. Microbiol. Mol. Biol. Rev. 74, 434–452. doi: 10.1128/mmbr.00020-10, PMID: 20805406 PMC2937521

[ref18] Sun J. Yang R. S. Zhang Q. Feng Y. Fang L. X. Xia J.. (2016). Co-transfer of *bla*_NDM-5_ and *mcr-1* by an IncX3-X4 hybrid plasmid in *Escherichia coli*. Nat. Microbiol. 1, 16176. doi: 10.1038/nmicrobiol.2016.176, PMID: 27668643

[ref19] Tang B. Chang J. Zhang L. Liu L. Xia X. Hassan B. H.. (2020). Carriage of distinct *mcr-1*-harboring plasmids by unusual serotypes of salmonella. Adv. Biosyst. 4:e1900219. doi: 10.1002/adbi.201900219, PMID: 32293141

[ref20] Wang C. Feng Y. Liu L. Wei L. Kang M. Zong Z. (2020). Identification of novel mobile colistin resistance gene *mcr-10*. Emerg. Microbes Infect. 9, 508–516. doi: 10.1080/22221751.2020.1732231, PMID: 32116151 PMC7067168

[ref21] Wang R. van Dorp L. Shaw L. P. Bradley P. Wang Q. Wang X.. (2018). The global distribution and spread of the mobilized colistin resistance gene *mcr-1*. Nat. Commun. 9, 1179. doi: 10.1038/s41467-018-03205-z, PMID: 29563494 PMC5862964

[ref22] Wang X. Wang Y. Zhou Y. Wang Z. Wang Y. Zhang S.. (2019). Emergence of Colistin resistance gene mcr-8 and its variant in *Raoultella ornithinolytica*. Front. Microbiol. 10, 228. doi: 10.3389/fmicb.2019.00228, PMID: 30828324 PMC6384272

[ref23] Wei D. W. Wong N. K. Song Y. Zhang G. Wang C. Li J.. (2022). IS26 veers genomic plasticity and genetic rearrangement toward carbapenem hyperresistance under sublethal antibiotics. MBio 13:e0334021. doi: 10.1128/mbio.03340-21, PMID: 35130728 PMC8822349

[ref24] Wong M. H. Chan E. W. Chen S. (2017). IS*26*-mediated formation of a virulence and resistance plasmid in Salmonella Enteritidis. J. Antimicrob. Chemother. 72, 2750–2754. doi: 10.1093/jac/dkx238, PMID: 29091201

[ref25] Wu R. Yi L. X. Yu L. F. Wang J. Liu Y. Chen X.. (2018). Fitness advantage of *mcr-1*-bearing Inc I2 and IncX4 plasmids in vitro. Front. Microbiol. 9:331. doi: 10.3389/fmicb.2018.00331, PMID: 29535696 PMC5835064

[ref26] Xu L. Wang P. Cheng J. Qin S. Xie W. (2019). Characterization of a novel *bla* _NDM-5_-harboring IncFII plasmid and an *mcr-1*-bearing IncI2 plasmid in a single *Escherichia coli* ST167 clinical isolate. Infect. Drug Resist. 12, 511–519. doi: 10.2147/idr.S192998, PMID: 30881056 PMC6402710

[ref27] Yang X. Xie M. Xu Q. Ye L. Yang C. Dong N.. (2022). Transmission of pLVPK-like virulence plasmid in *Klebsiella pneumoniae* mediated by an Incl 1 conjugative helper plasmid. iScience 25:104428. doi: 10.1016/j.isci.2022.104428, PMID: 35663037 PMC9160755

[ref28] Ye H. Li Y. Li Z. Gao R. Zhang H. Wen R.. (2016). Diversified *mcr-1*-Harbouring plasmid reservoirs confer resistance to colistin in human gut microbiota. MBio 7:e00177. doi: 10.1128/mBio.00177-16, PMID: 27048797 PMC4817250

