# Supplementary figures and images for: Emergence of a novel hybrid mcr-1-bearing plasmid in an NDM-7-producing ST167 Escherichia coli strain of clinical origin
Source: Front Microbiol. 2022 Aug 24;13:950087. doi: 10.3389/fmicb.2022.950087 (PMC9449459; doi:10.3389/fmicb.2022.950087)

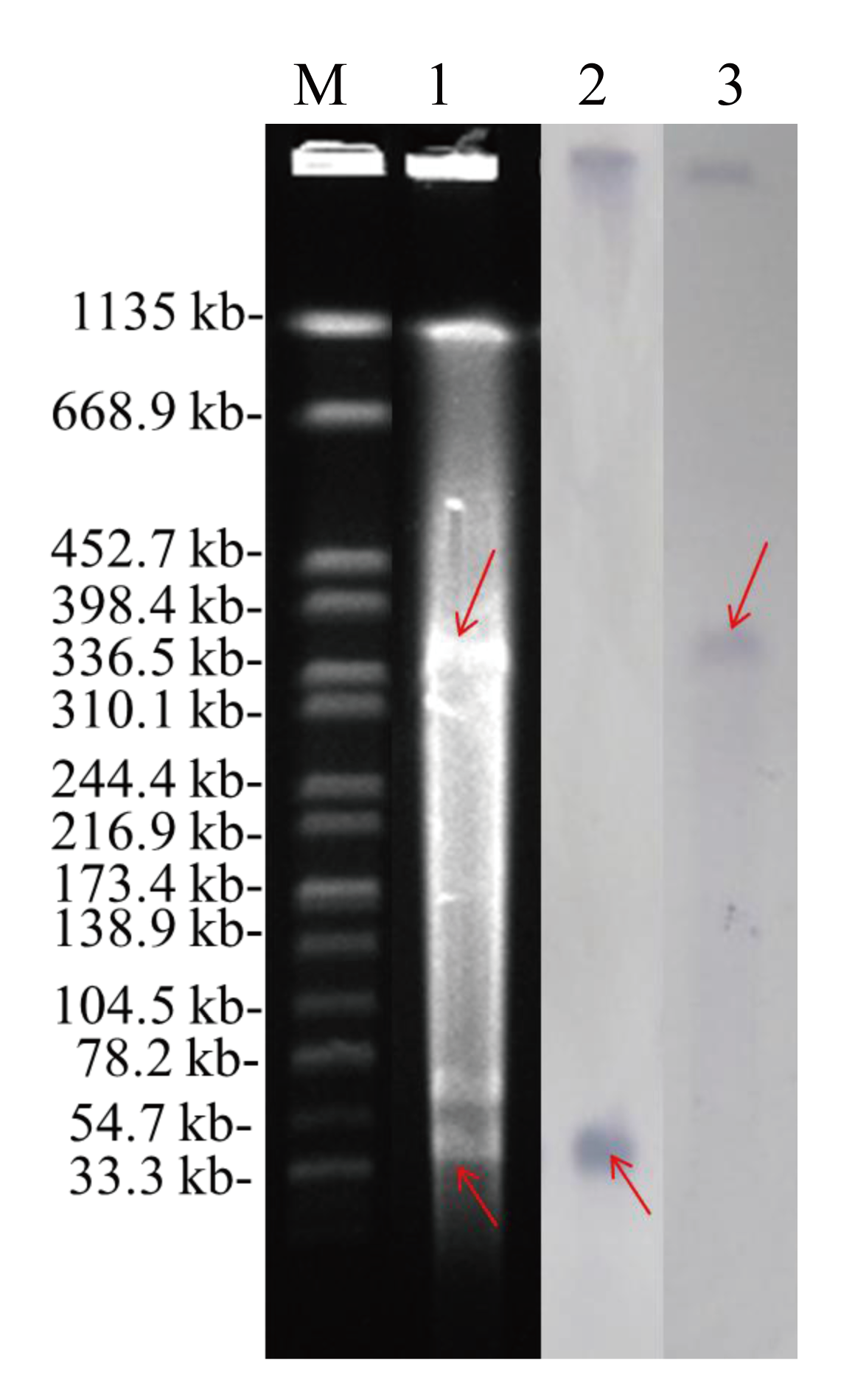

Supplement: Supplementary file 1 [file Data_Sheet_1.ZIP › Supplementary Material Presentation/Figure 1.tif]

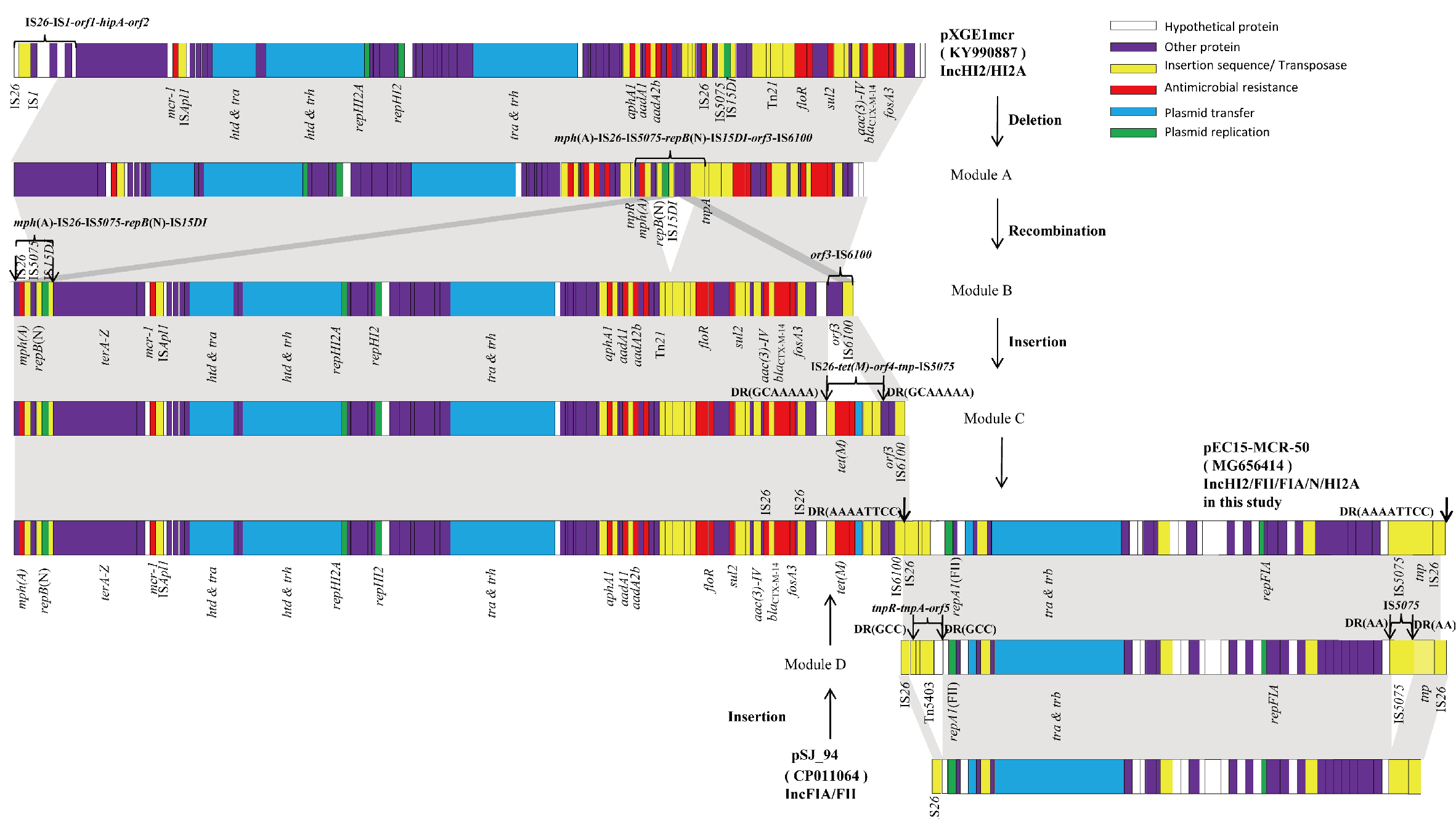

Supplement: Supplementary file 1 [file Data_Sheet_1.ZIP › Supplementary Material Presentation/Figure 2.ú¿Aú⌐.tif]

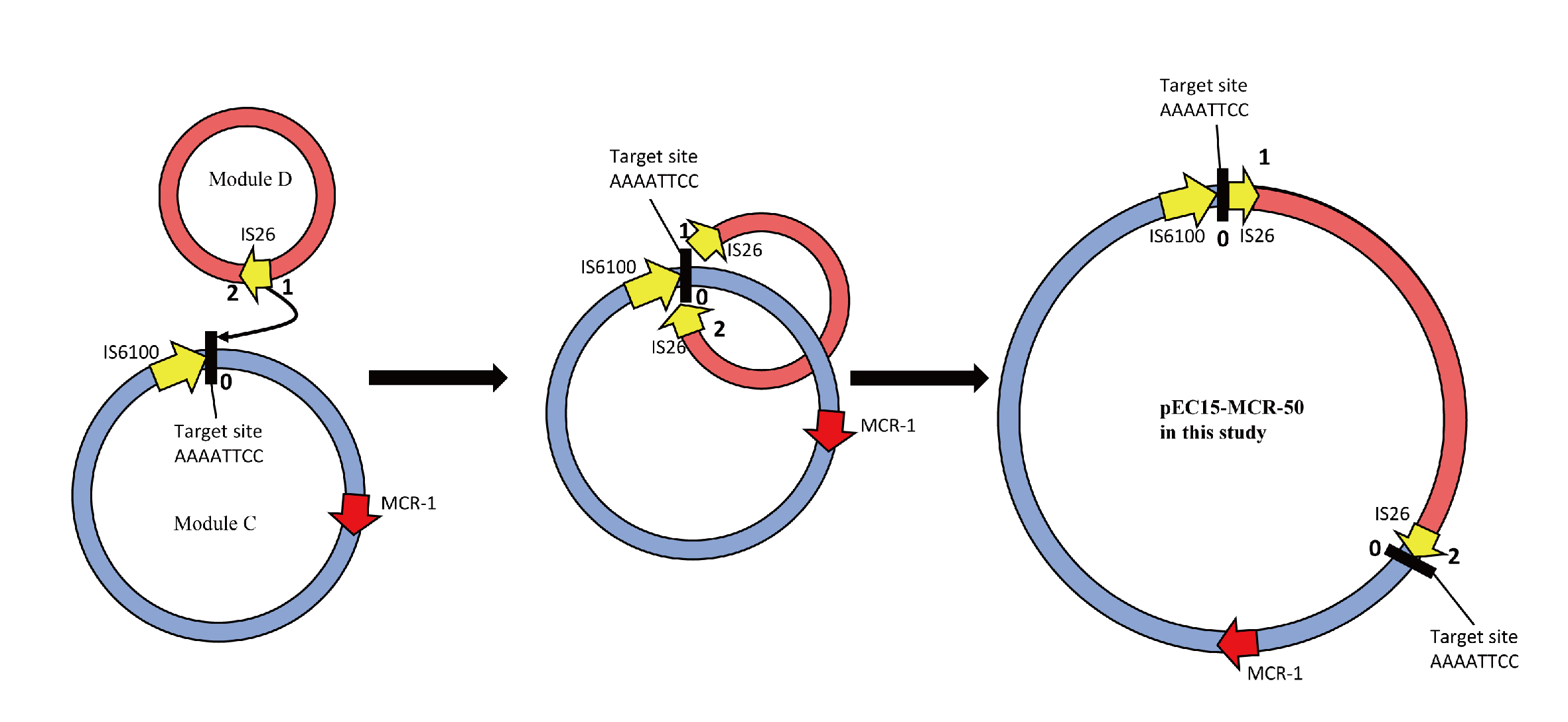

Supplement: Supplementary file 1 [file Data_Sheet_1.ZIP › Supplementary Material Presentation/Figure 2.ú¿Bú⌐.tif]
