# Supplementary material for: Emergence of a novel hybrid mcr-1-bearing plasmid in an NDM-7-producing ST167 Escherichia coli strain of clinical origin
Source: Front Microbiol. 2022 Aug 24;13:950087. doi: 10.3389/fmicb.2022.950087 (PMC9449459; doi:10.3389/fmicb.2022.950087)
Supplement: Supplementary file 2 [file Table_1.DOCX]

[**Table S1**](https://www.frontiersin.org/articles/10.3389/fmicb.2021.626160/full#S10)**.** PCR primers used for detecting the structure of pEC15-MCR-50 after passage.

| Primers | Sequence（5‘-3’） | Annealing Temperature（℃） | Product  Size（bp） |
| --- | --- | --- | --- |
| F1 | GGGGATCACACCCCATATG | 60 | 2984 |
| R1 | GGATCATTGAGACGCGTTAC |  |  |
| F2 | AAGAATCTGACCACGTGGA | 58 | 2586 |
| R2 | TGATGGCCTTGACAACCAAG |  |  |
| F3 | CCAAGGAGAGCTCATGAATG | 60 | 1850 |
| R3 | GGGTTTCCATTCGAGAAGAAA |  |  |
| F4 | GAGAACCAGCCAATTGATCT | 62 | 1811 |
| R4 | GTAAGAATTCTGAAGACAGACGC |  |  |
| F5 | GTTCCGATTGCTCACATTTT | 56 | 2539 |
| R5 | GGTGATCAGTAGCATCGC |  |  |
| F6 | GCGGTCAATTACGCCAGA | 58 | 2216 |
| R6 | CAGCAAGTAGGCGTTTATTTG |  |  |
| F7 | AGTTTGCCAAATTCACGC | 54 | 3301 |
| R7 | CAGTGGGCTTGGCTTATG |  |  |
| F8 | CTTCAAGATGCCCCATAAACG | 59 | 2794 |
| R8 | GAACGCGCCTTTATTGATG |  |  |
| F9 | GGCGTCCGTTAGTTTTCAAC | 59 | 3050 |
| R9 | ATTATGCGGCTTAAAGAGCG |  |  |
| F10 | TATATCGCCGCTTTCGGTG | 60 | 2088 |
| R10 | GGTATTCCTGATGACTGATGG |  |  |
| F11 | TTCCTCGTACGGCTGGTAC | 61 | 1441 |
| R11 | AAGTGATCGGTCTTGAAGACC |  |  |
